# Genomic features of renal cell carcinoma with venous tumor thrombus

**DOI:** 10.1038/s41598-018-25544-z

**Published:** 2018-05-10

**Authors:** Gregor Warsow, Daniel Hübschmann, Kortine Kleinheinz, Cathleen Nientiedt, Martina Heller, Laura Van Coile, Yanis Tolstov, Lukas Trennheuser, Kathrin Wieczorek, Carine Pecqueux, Claudia Gasch, Timur Kuru, Joanne Nyarangi-Dix, Gencay Hatiboglu, Dogu Teber, Sven Perner, Albrecht Stenzinger, Wilfried Roth, Boris Hadaschik, Sascha Pahernik, Dirk Jäger, Carsten Grüllich, Anette Duensing, Roland Eils, Matthias Schlesner, Holger Sültmann, Markus Hohenfellner, Stefan Duensing

**Affiliations:** 1 Division of Theoretical Bioinformatics (B080), German Cancer Research Center (DKFZ), Im Neuenheimer Feld 280, D-69120 Heidelberg, Germany; 2 Department for Bioinformatics and Functional Genomics, Institute for Pharmacy and Molecular Biotechnology (IPMB) and BioQuant, Heidelberg University, Im Neuenheimer Feld 267, D-69120 Heidelberg, Germany; 3 Department of Pediatric Immunology, Hematology and Oncology, University Hospital Heidelberg, Im Neuenheimer Feld 430, D-69120 Heidelberg, Germany; 4 Section of Molecular Urooncology, Department of Urology, University of Heidelberg School of Medicine, Im Neuenheimer Feld 517, D-69120 Heidelberg, Germany; 5 Department of Medical Oncology, University of Heidelberg School of Medicine, National Center for Tumor Diseases (NCT), Im Neuenheimer Feld 460, D-69120 Heidelberg, Germany; 6 Center for Kidney Tumors, National Center for Tumor Diseases and University of Heidelberg School of Medicine, Im Neuenheimer Feld 460, D-69120 Heidelberg, Germany; 7 Department of Urology, University of Heidelberg School of Medicine, Im Neuenheimer Feld 110, D-69120 Heidelberg, Germany; 8 Institute of Pathology, University of Heidelberg School of Medicine, Im Neuenheimer Feld 224, D-69120 Heidelberg, Germany; 9 Pathology of the University Hospital Schleswig-Holstein, Campus Lübeck and the Research Center Borstel, Leibniz Lung Center, Ratzeburger Allee 160, D-23538 Lübeck and Parkallee 1-40, D-23845 Borstel, Germany; 10 University of Pittsburgh Cancer Institute, Cancer Therapeutics Program, 5117 Centre Avenue, Pittsburgh, PA 15213 USA; 11 National Center for Tumor Diseases, German Cancer Research Center, Cancer Genome Research, Im Neuenheimer Feld 460 and German Cancer Consortium (DKTK), D-69120 Heidelberg, Germany; 12 Present Address: Department of Urology, University of Cologne, Kerpener Str. 62, D-50937 Cologne, Germany; 13 Present Address: Institute of Pathology, University of Mainz Medical School, Langenbeckstr. 1, D-55131 Mainz, Germany; 14 Present Address: Department of Urology, University Hospital Essen, University of Duisburg-Essen, Hufelandstr. 55, D-45122 Essen, Germany; 15 Present Address: Department of Urology, Nuremberg Hospital, Paracelsus Medical University, Prof.-Ernst-Nathan-Strasse 1, D-90419 Nuremberg, Germany; 16 Present Address: Bioinformatics and Omics Data Analytics (B240), German Cancer Research Center (DKFZ), Im Neuenheimer Feld 280, D-69120 Heidelberg, Germany

## Abstract

A venous tumor thrombus (VTT) is a potentially lethal complication of renal cell carcinoma (RCC) but virtually nothing is known about the underlying natural history. Based on our observation that venous thrombi contain significant numbers of viable tumor cells, we applied multiregion whole exome sequencing to a total of 37 primary tumor and VTT samples including normal tissue specimens from five consecutive patients. Our findings demonstrate mutational heterogeneity between primary tumor and VTT with 106 of 483 genes (22%) harboring functional SNVs and/or indels altered in either primary tumor or thrombus. Reconstruction of the clonal phylogeny showed clustering of tumor samples and VTT samples, respectively, in the majority of tumors. However, no new subclones were detected suggesting that pre-existing subclones of the primary tumor drive VTT formation. Importantly, we found several lines of evidence for “BRCAness” in a subset of tumors. These included mutations in genes that confer “BRCAness”, a mutational signature and an increase of small indels. Re-analysis of SNV calls from the TCGA KIRC-US cohort confirmed a high frequency of the “BRCAness” mutational signature AC3 in clear cell RCC. Our findings warrant further pre-clinical experiments and may lead to novel personalized therapies for RCC patients.

## Introduction

Metastatic renal cell carcinoma (RCC) is the most lethal urological cancer and characterized by extensive genomic intratumoral heterogeneity^1,2^. In approximately 4–25% of RCC patients, the primary tumor extends into the renal vein, the inferior vena cava or as far as the right atrium of the heart, usually under formation of a large blood clot^3–5^. RCC tumor thrombi are subdivided macroscopically according to their consistency into solid or friable. The latter has been suggested to be associated with a worse prognosis although this notion has recently been challenged^6–8^. Despite the poor prognosis associated with venous tumor thrombus (VTT) formation if left untreated^9–11^, virtually nothing is known about its natural history.

Clear cell RCC has previously been shown to exhibit a high degree of mutational heterogeneity and patterns of branched and convergent clonal evolution^1,2^. There is evidence that functional and genomic intratumoral heterogeneity in RCC is shaped by spatial niches^12,13^. To what extent these findings apply to RCC with VTT is currently not known in detail^14^.

Herein, we used multiregion whole exome sequencing of RCCs with VTT to characterize the mutational landscape of these tumors. We found heterogeneous mutations between primary tumor and VTT, however, our results suggest that pre-existing subclones of the primary tumor drive VTT formation. Importantly, we provide evidence for homologous recombination defects (“BRCAness”) in a subset of RCCs with VTT, which holds the promise for novel personalized therapeutic interventions in patients with this condition including PARP inhibitors, platinum salts or others^15^.

## Methods

### Patients

A total of five consecutive patients with RCC and venous tumor thrombus were prospectively included after written informed consent and under Ethics committee vote 207/2005 of the University of Heidelberg School of Medicine. All patients showed a clear cell histomorphology except patient RCC-VTT-04 with a papillary type II RCC.

### Whole exome sequencing

DNA extraction and whole exome sequencing were performed by GATC Biotech (Konstanz, Germany) on an Illumina platform (InView™ Human Exome). Fastq files were aligned against the human reference genome (build 37, version hs37d5), using bwa mem (version 0.7.8 with minimum base quality threshold set to zero [−T 0] and remaining settings left at default values), followed by coordinate-sorting with bamsort (with compression option set to fast (1)) and marking duplicate read pairs with bammarkduplicates (with compression option set to best (9)) (both part of the biobambam package version 0.0.148).

The generated bam files were used to identify high-confidence somatic SNVs by applying an in-house analysis pipeline based on samtools^16^ and bcftools (both version 0.1.19) as described earlier^17,18^. Briefly, samtools version 0.1.19^16^ was used for calling all candidate variants (positions where the tumor sample differs from the reference sequence). The filter, which usually makes samtools a germline caller, was switched off. Candidate variants were postfiltered by custom filters optimized for somatic variant calling. The discrimination between somatic and germline variants was performed by looking up the variant position in the matched normal sample.

Results were amended by additional classification of the SNVs into 96 categories according to their triplet of the base preceding the SNV, the actual transition/transversion and the following base. For each class it was determined whether its SNVs exhibit a sequencing strand bias. For classes with bias, SNVs with less than two supporting reads on the strand opposite to the strand exhibiting the biased variants were filtered out. Somatic SNVs were called functional, when they were nonsynonymous, occurred within splice sites, or lead to gain or loss of stop codons. All SNVs were annotated using Annovar^19^ (version as of February 2016), dbSNP (build 147)^20^, 1000 Genomes^21^ and EVS (Exome Variant Server, NHLBI GO Exome Sequencing Project (ESP), Seattle, WA [version ESP6500SI-V2]) data. Indels were identified with Platypus callVariants (version 0.8.1, parameter settings genIndels = 1, genSNPs = 1, bufferSize = 100000 and maxReads = 10000000)^22^. All indels were annotated using Annovar^19^ (version as of November 2014), dbSNP (build 141)^20^, 1000 Genomes^21^ data.

Sequence data for this study is available in the European Genome-phenome Archive (EGA), which is hosted by the EBI, under accession number EGAS#00001001950.

### Supervised Analysis of Mutational Signatures

A supervised analysis of mutational signatures determines contributions of known mutational signatures to the mutational catalogue, i.e. counts of nucleotide exchanges in their motif context, of a given dataset. As opposed to an unsupervised analysis of mutational signatures, i.e. a discovery method like non-negative matrix factorization (NMF) as used in^23^, a supervised analysis requires much less statistical power, but will not discover new signatures. A software to carry out such an analysis was written as an R package (*YAPSA* – *Yet Another Package for Signature Analysis*^24^; manuscript in preparation). Due to the low requirements on statistical power, i.e. the number of samples in a cohort and the number of point mutations per sample, the supervised analysis of mutational signatures as carried out by *YAPSA* may be run on very small cohorts.

Using *YAPSA*, a linear combination decomposition of the mutational catalogue with known and predefined signatures was computed by non-negative least squares (NNLS), functions for which are implemented in the R package *lsei*^25^. In order to increase specificity, the NNLS algorithm was applied twice: after the first execution, only those signatures whose exposures, i.e., contributions in the linear combination, were higher than a certain cutoff, were kept and the NNLS was run again with the reduced set of signatures. As detectability of the different signatures may vary, the cutoffs were chosen to be signature-specific.

In the analysis presented here, we used a set of 30 publicly available mutational signatures (http://cancer.sanger.ac.uk/cosmic/signatures), which were identified in an unsupervised analysis of 10,952 whole exome sequenced and 1,048 whole genome sequenced tumor normal control pairs (12,000 pairs in total). We call these signatures, which are an extension of the signatures published initially in^23^, AC1 – AC30 (AC standing for *Alexandrov COSMIC*). The signature-specific cutoffs were determined in a receiver operator characteristic (ROC) analysis using publicly available data on mutational catalogues of the samples included in the initial Alexandrov analysis^23^, consisting of 7,042 tumor normal control pairs (507 from whole genome sequencing and 6,535 from whole exome sequencing).

Before running an analysis, *YAPSA* builds up the mutational catalogues of the samples or cohorts to be analyzed by calling the functions *mutationContext* and *mutationContextMatrix* from the R package *SomaticSignatures*^26^. The mutational catalogue was then corrected for different occurrences of the motifs between the whole genome and the target capture used for whole exome sequencing of the tumor of our patients.

### Copy number alterations (CNAs), tumor cell content (TCC) and ploidy estimation

CNAs were inferred from whole exome sequencing data with cnvKit version 0.8.6 (git repository hash: 85774ac)^27^ with default parameter settings.

Heterozygous SNPs were determined as those positions with alternative allele fraction between 0.3 and 0.7 in the respective normal sample. Segments which contained at least 20 heterozygous SNPs were further processed to infer sample ploidy and tumor cell content along with allele-specific copy number estimates. The segments were classified as balanced or imbalanced according to the distribution of the frequencies of the alternative allele of the SNPs in the respective segments. If this distribution had a global maximum between 0.45–0.55 a segment was called balanced, remaining segments were further separated into two groups – ambiguous segments with one density peak outside of the above mentioned interval, and imbalanced segments with two peaks. Ambiguous segments were neglected in subsequent steps. For imbalanced segments, the mean B-allele frequency (BAF) of all SNPs in the segment that were heterozygous in the germline was estimated using the allele with the higher read count as B-allele. Then the mean read count of the B-allele was calculated as product of total coverage and the BAF of the respective segment.

Tumor cell content (TCC) and ploidy of a sample were estimated using a method adapted from ACEseq (manuscript in preparation). For TCC estimation values in the range of 0.15–1.0 were tested whereas a ploidy range between 1 and 6.5 was allowed. For each possible combination of TCC and ploidy, absolute copy numbers as well as allele-specific copy numbers and the decrease in heterozygosity (DH)^28^ were estimated segment-wise. Allele-specific copy numbers were calculated as total copy number divided by two for balanced segments and as a function of coverage and B-allele read counts in case of imbalanced segments. The weighted mean distance of all segments to the next allowed integer copy number state was calculated for total and allele-specific copy numbers. Here, allowed means even total copy number states for balanced segments and any integer copy number state for imbalanced segments and allele-specific copy numbers. TCC/ploidy combinations requiring negative copy number states or a DH larger than 1 for any segment were excluded. Local minima in the weighted mean distance were considered as possible TCC/ploidy solution for the sample and were visually evaluated. Additionally, TCC was estimated from the mutant allele fraction distribution of somatic SNVs and CNV-based and SNV-based estimates were compared.

Due to low TCC, resulting in a weak signal for imbalance detection, some samples required manual adjustments. For the sample RCC-VTT-01 thrombus III (7), regions that were erroneously annotated as balanced by the automated detection were reclassified as imbalanced based on the coverage profile and comparison to the remaining samples from the same patient. B-allele read counts were recalculated for these segments and a rerun of ploidy and TCC estimation was performed. All samples from patients RCC-VTT-02 and RCC-VTT-03 showed a low TCC. Only one sample from RCC-VTT-02 (tumor (1)) had sufficient TCC for proper calling of allelic states. For all other RCC-VTT-02 samples the restrictions for balanced segments were loosened so they could be fitted to uneven copy numbers too to account for undetected imbalances. This lead to a better fit of the copy numbers though TCC was still estimated too high based on visual inspection of the BAF profiles and compared to the MAF estimates, most likely due to high subclonality or low signal due to the low TCC. RCC-VTT-02 tumor (1) was used as reference for the newly obtained calls to evaluate the allele-specific estimates as some of its CNAs were also expected to appear in the other samples with lower purity. The new ploidy fits were combined with TCCs that were based on the MAF estimates. Good results could be obtained with this approach with respect to small deviations from integer copy number states and overall comparison against RCC-VTT-02 tumor (1). For patient RCC-VTT-03, all samples showed low TCC (<30%, based on MAF estimates and visual inspection of BAF profiles), so no reliable estimates for balanced and imbalanced states could be achieved. As we were missing a reference for this case, no allele specific copy numbers could be obtained for this patient. Similar to RCC-VTT-02, TCC was based on the MAF distribution whereas the ploidies were estimated with the constraint that the lowest observed coverage had to correspond to one copy and the majority of the genome fitted to a diploid state.

### HRD (Homologous Recombination Deficiency) and LST (Large Scale Transition) scores estimation

For stable HRD and LST scores estimation, copy number profiles were smoothed to reduce oversegmentation caused by technical noise. All neighboring segments that rounded to the same total and allele specific copy number and did not deviate by more than 0.3 from another were merged. In addition to smoothing of similar segments any segments smaller than 3 Mb were merged with their more similar neighbor as suggested by Popova *et al*.^29^.

Any switch between copy number states of segments that were larger than 10 Mb and did not correspond to full chromosome arms was counted as LST according to Popova *et al*.^29^. Additionally, segments that were larger than 15 Mb but less than a whole chromosome in length and corresponded to a loss of heterozygosity (LOH) were counted for the HRD estimation^30^.

In case of RCC-VTT-03, only segments with a single copy were taken into account for HRD estimation due to the lack of allele-specific copy numbers.

### Heterogeneity analysis

In order to reconstruct the clonal evolution of the tumor samples, the R package Canopy version 1.1.1 was used^31^. Canopy takes two sources of information as input: MAF values of SNVs and CNAs. In this project, functional somatic SNVs and smoothed copy number segments (as described above) were used. Segments, which were shared by all samples were removed, as they do not contribute to discrimination between samples. Additionally, according to the advice of the author of the software, small segments (<1 kbp) were neglected, as larger events tend to be more reliable than small ones, especially on exome sequencing data. For each patient, the optimal number of subclones K was identified by Canopy based on the Bayes Information Criterion (BIC), followed by a Markov chain Monte Carlo (MCMC) based approach to determine a tree topology with K leaves and a mutation setting in this tree, which explains the observed mutation pattern with highest likelihood. MCMC sampling steps were performed over 50,000 iterations in 30 individual chains (to avoid local optima) in a parallelized manner. Mutations defining individual subclones are available upon request.

### Immunohistochemistry

Formalin-fixed, paraffin-embedded tissue specimens were provided by the tissue bank of the National Center for Tumor Diseases (NCT, Heidelberg, Germany) in accordance with the regulations of the tissue bank and the approval of the ethics committee of the University of Heidelberg School of Medicine. Paraffin sections were deparaffinized in xylene and rehydrated in a graded ethanol series. Antigen retrieval was performed with a steam cooker using retrieval buffer (Target Retrieval Solution, Dako). Primary antibodies used were directed against Ki-67 (clone MIB-1, Dako, 1:100), phospho-S6 ribosomal protein S235/236 (Cell Signaling; 1:100) or γH2AX (clone JBW301, Millipore, 1:100) and were incubated overnight at 4 °C. Immunodetection was performed using the Histostain-Plus Detection Kit (3^rd^ Generation, Invitrogen) according to manufacturer’s recommendations. Nuclear counterstaining was provided by hematoxylin (Thermo Scientific). Tissue specimens were analyzed by two independent observers (L.V.C., M.He.). For statistical analysis, the Mann Whitney U test, Wilcoxon rank-sum test or Student’s t test for independent samples were used wherever applicable. Differences with a p value of ≤0.05 were considered significant.

All methods were performed in accordance with the relevant guidelines and regulations and experimental protocols were approved by the Ethics committee of the University of Heidelberg School of Medicine (206/2005). Datasets generated during the current study and not deposited at EGA are available from the corresponding author at reasonable request.

### Somatic variant calls in the TCGA KIRC-US cohort

Somatic SNV calls from the TCGA KIRC-US cohort^32^ were downloaded from https://portal.gdc.cancer.gov/legacy-archive/files/3e93f2c1–559f-4747-9c53-30589dbc03f7 as per February 5^th^, 2018. Samples were filtered for mutational load and only those with more than 25 somatic SNVs were kept for the analysis, leaving 400 samples for the analysis. In complete analogy to the analysis of mutational signatures of our own samples, a mutational catalog was built, correction for triplet content was performed and a supervised analysis of mutational signatures using YAPSA^24^ was performed.

## Results

### Venous tumor thrombi contain viable and proliferating RCC cells

**Figure 1:**
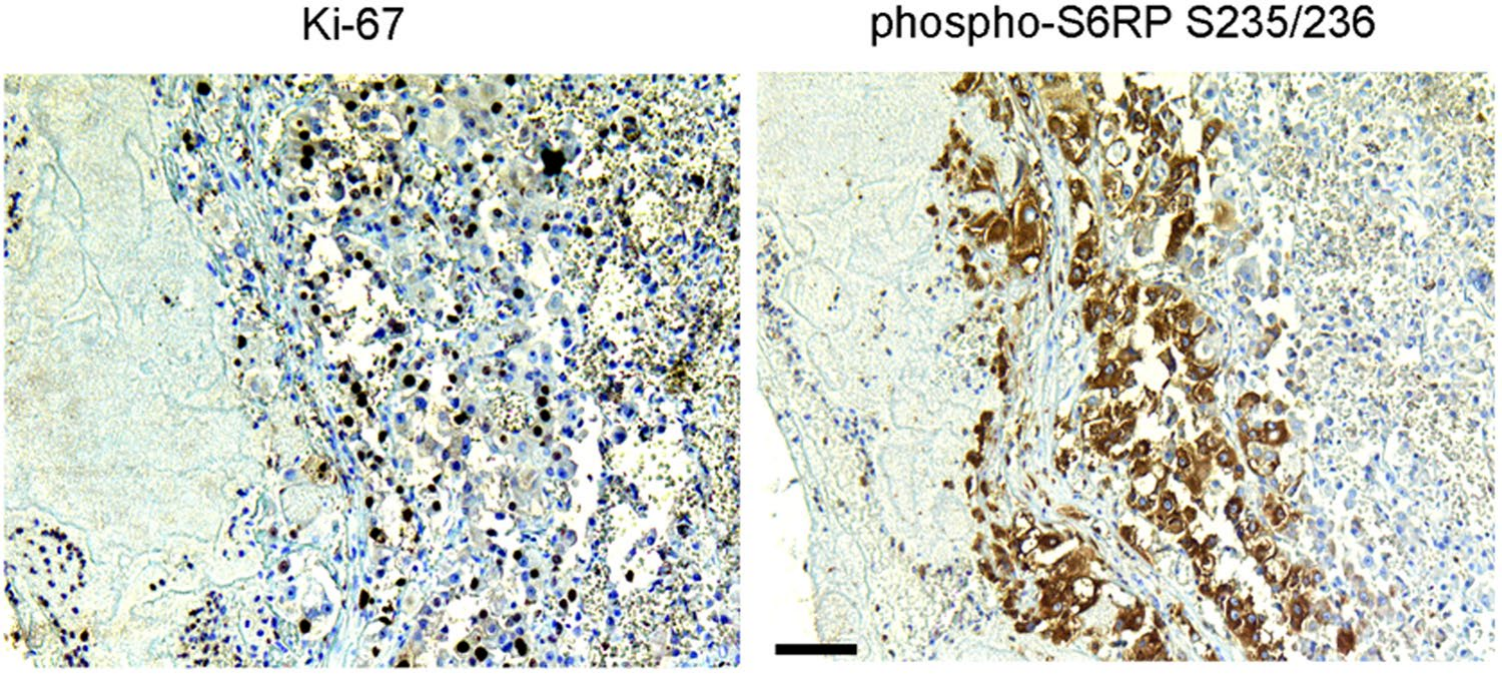
Tumor thrombi contain vital tumor cells. Representative immunohistochemical staining of a venous tumor thrombus for Ki-67 and phospho-S6RP S235/236. The tumor was unrelated to the five RCC-VTT cases analyzed. Scale bar = 100 μm.

In exploratory experiments that preceded the multiregion whole exome sequencing study, we analyzed representative samples from 14 RCCs with VTT to assess if viable cells were present in the blood clot (Fig. 1). Unexpectedly, we detected a considerable number of proliferating Ki-67-positive tumor cells in the venous thrombi (mean 25.6 cells per 40× high-power field [HPF]; range, 2.3–88.7 cells/HPF). Moreover, we found tumor cells showing activated growth and survival signaling via the PI3K-AKT-mTOR axis as evidenced by positivity for phospho-S6RP S235/236 (mean 4.8 cells per HPF; range 0–11.9 cells/HPF).

### Mutational landscape of RCC with VTT

**Figure 2:**
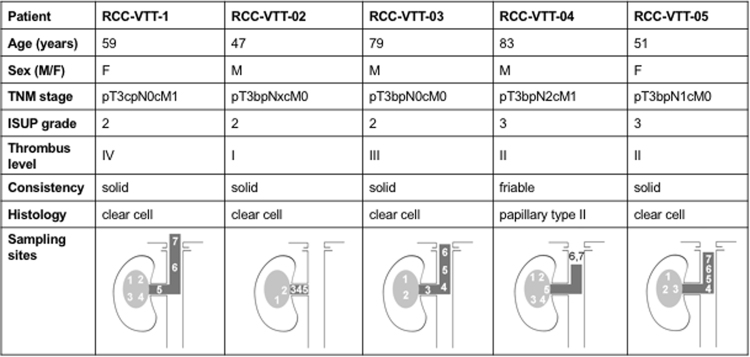
Patient characteristics and sampling sites. VTT levels are indicated according to Novick^48^.

Encouraged by the finding that venous thrombi contain a substantial amount of viable tumor cells, we performed multiregion whole exome sequencing of a total of 37 samples from five consecutive patients with RCC and VTT (Fig. 2) to directly compare the mutational landscape between the primary tumor and the corresponding VTT. Four patients were diagnosed with clear cell renal cell carcinoma whereas one patient (RCC-VTT-04) had a poorly differentiated type II papillary RCC and, in line with previous reports, mutations in *NF2* and *SETD2* (see below)^33,34^. The latter patient had a friable tumor thrombus, whereas the other four were of solid consistency (Fig. 2).

DNA isolated from normal tissue (n = 5), different locations of the primary tumors (n = 16) and different levels of venous tumor thrombi (n = 16; Fig. 2) were sequenced to >35-fold target coverage (mean target coverage: 48.7× [range: 37×– 99.5×]; Suppl. Table 1). In all patients, the minor allele frequency (MAF) distribution was comparable between samples from the primary tumor and VTT indicating that results were not due to an uneven admixture of non-tumorous cells (Suppl. Fig. 1).

**Figure 3:**
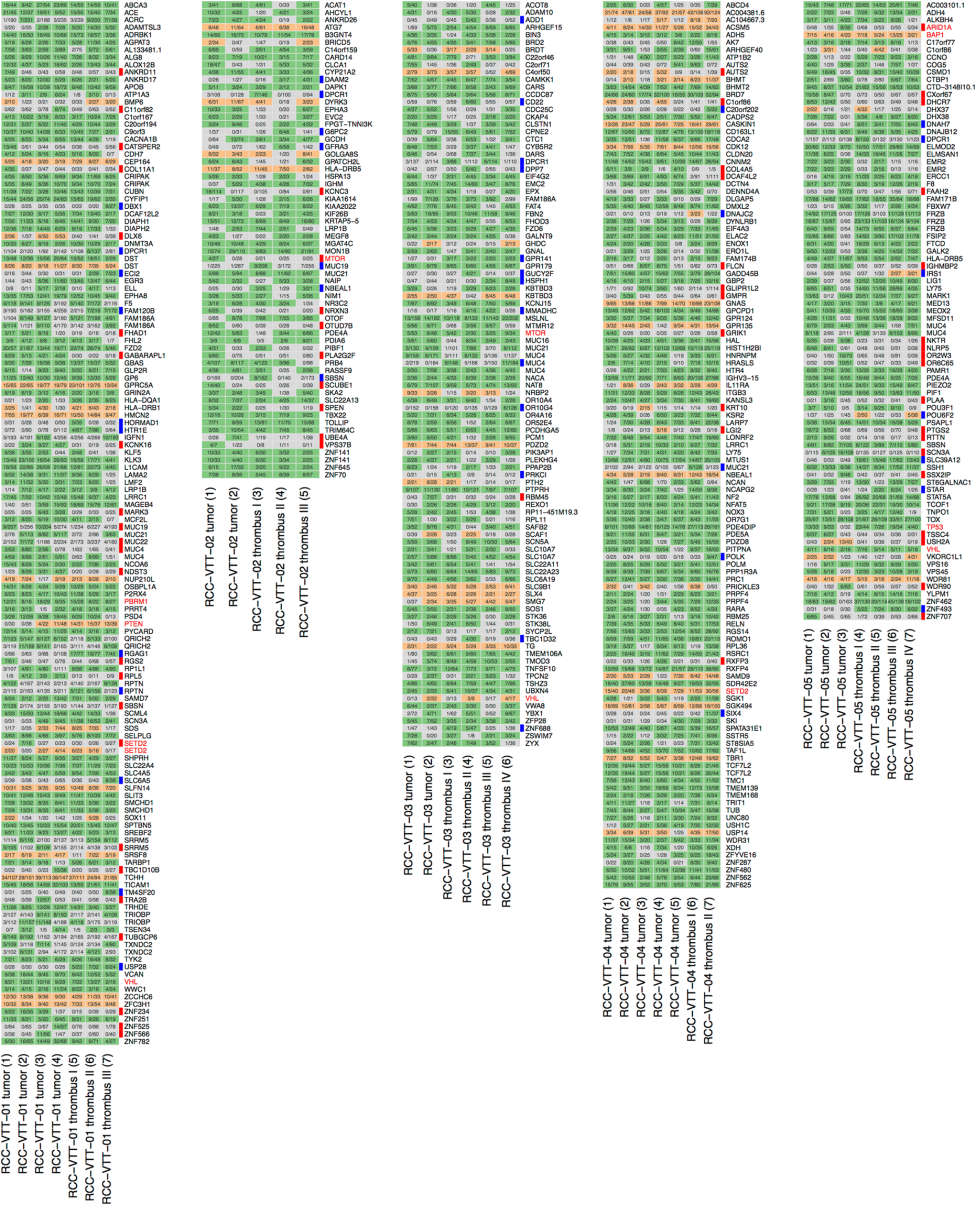
Mutational landscape of RCC with venous tumor thrombus. Functional SNVs (green) and indels (orange) in 483 genes are shown (grey indicates absence of a mutation). Numbers in cells describe the number of reads carrying the alternative allele and the respective sequencing coverage at this position. Bars on the right side of each plot indicate primary tumor-specific (red) or VTT-specific (blue) SNVs and indels. Driver mutations are highlighted in red.

Across all samples, we identified 442 somatic functional SNVs and 64 functional indels in a total of 483 genes (Suppl. Table 2). Known RCC-associated genes and drivers were mostly found to be mutated in both the primary tumor and the VTT (*VHL*, *PBRM1*, *SETD2*, *BAP1*, *PTEN*, *MTOR* and *TP53*) with the exception of an *MTOR* mutation in RCC-VTT-02 and an *ARID1A* mutation in RCC-VTT-05 (Fig. 3)^32^. Among the genes mutated, a number of DNA damage checkpoint and repair genes stood out including *BAP1*, *CDK12*, *CEP164*, *ERCC1*, *LIG1*, *POLK*, *POLM* and *USP28* (Fig. 3)^35–37^.

Overall, 106 of 483 genes (22%) harboring functional SNVs and/or indels were heterogeneous between primary tumor and thrombus i.e., they were altered only in one but not the other of the two compartments (Suppl. Table 2 and Fig. 3). The sum of the number of genes that were mutated exclusively in the primary tumor (by somatic SNVs and/or indels) but not the VTT of the same patient was 63 (13% of all altered genes; patient-individual numbers ranged from 1 to 24) across all patients. Conversely, a total of 43 genes (8.9% of all altered genes) were mutated exclusively in venous tumor thrombi but not in the corresponding primary tumor (range from 5 to 13; Suppl. Table 2).

Copy number alterations (CNAs) inferred from exome sequencing data after correction for purity showed a loss of chromosome 3p except in patient RCC-VTT-04 with a papillary type II RCC (Suppl. Fig. 2).

Taken together, these results underscore that venous thrombi in RCC contain viable tumor cells with heterogeneous mutations, some of which affect genes involved in the cellular response to DNA damage.

### Clonal phylogeny in RCC with VTT

**Figure 4:**
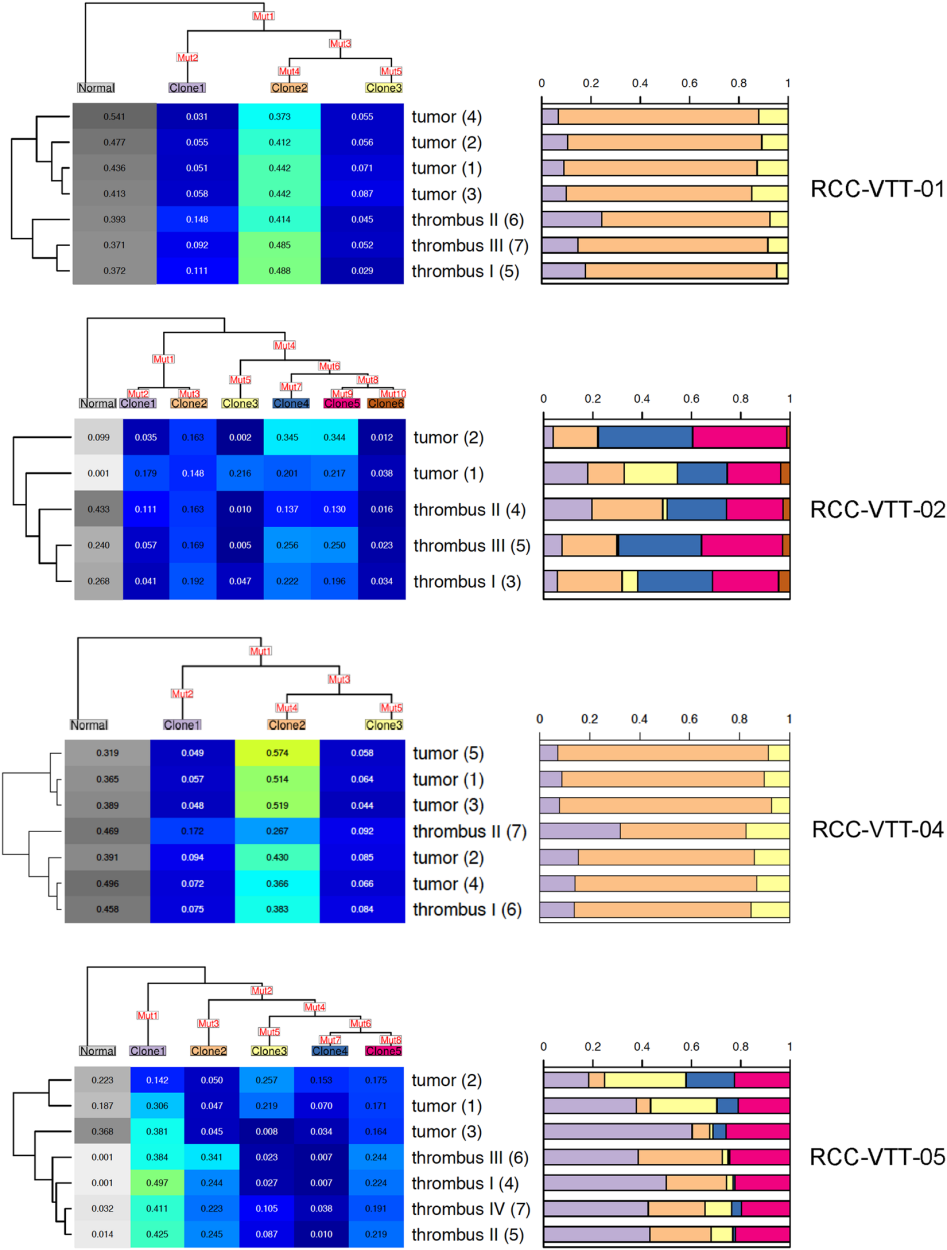
Clonal evolution in RCC with VTT. The clonal heterogeneity for all samples was determined using Canopy. Clonal evolution is depicted as a tree, the leaves of which correspond to the subclones denoted by “Clone1” up to “Clone6”. The number of leaves corresponds to the optimal number of subclones. The set of all mutations (SNVs and CNAs) among all samples of the patient is distributed along the tree in a (heuristically) optimal way (“Mut1”–“Mut10”). Each clone carries all the mutations, which can be collected when following the path from the respective leaf to the root of the tree. The matrix below the mutation tree shows the subclonal composition of each sample (values per line add up to 1). The leaf named “Normal” represents normal tissue contamination and has been neglected in further analyses. The contributions of each other subclone to a sample are illustrated in a stacked bar plot to the right of the matrix, the colors of which correspond to the colors of the leaf labels of the mutation tree. To the left of the matrix, the relationship between the samples with respect to subclonal composition (without “Normal”) is illustrated by a dendrogram.

We next sought to analyze the relationship between primary tumor and VTT in greater detail. To reconstruct the phylogeny of the tumor samples, the R package Canopy was used^31^. With the help of Canopy, we attempted to reconstruct the subclonal evolution of the tumor and subclone contributions to the individual primary tumor and VTT samples. Canopy was applied to the patients individually. As input for Canopy, we took the smoothed copy number segment data and the collection of all SNVs which were identified as somatic and functional in any of the samples of the respective patient. As shown in Fig. 4, this approach was able to model the clonal evolution and waves of mutational events for four of the five tumors with time running down vertically from the root to the bifurcations of the phylogenetic tree. RCC-VTT-03 was excluded from the analysis with Canopy because of insufficient purity. Each sample consisted of a mixture of subclones that are represented as stacked bar plots (Fig. 4, right panels).

In three of the four tumors that entered the final analysis, there was a phylogenetic separation between primary tumor and VTT (Fig. 4, dendrograms on the left) suggesting an evolutionary distance between the two compartments. There were also noticeable differences in the number of subclones and subclonal composition modeled by Canopy. Whereas SNVs and CNAs in RCC-VTT-01 and -04 were modeled into three subclones with a relatively even admixture in primary tumor and VTT, RCC-VTT-02 and -05 showed a higher number of subclones (up to six) and greater differences in the subclonal composition of a given sample.

We next analyzed whether the higher subclonal heterogeneity detected in RCC-VTT-02 and -05 may be associated with enhanced replication stress, a major cause of genomic instability and clonal heterogeneity^38^. An immunohistochemical analysis of corresponding primary tumor and thrombus of the five sequenced patients for proliferative activity (Ki-67) and signs of an activated DNA damage response (γH2AX; Suppl. Fig. 3) was performed. We found a consistently and significantly (p values between p ≤ 0.05 and p ≤ 0.0005) higher proliferation rate in the venous thrombi in comparison to at least one of the respective primary tumor samples (Suppl. Fig. 3). At the same time, tumor cells with γH2AX foci indicating an activated DNA damage response were detected in all primary RCCs and corresponding venous tumor thrombi at varying levels (Suppl. Fig. 3). Although a high number of γH2AX foci-positive tumor cells were detected in RCC-VTT-05, which also showed a high clonal diversity, there were overall no fundamental differences between tumors with high clonal diversity in comparison to RCCs with lower clonal diversity. This finding suggests that replication stress may not play a major role in shaping clonal heterogeneity.

Taken together, these findings indicate that a phylogenetic separation of tumor and VTT can occur without new mutations suggesting that pre-existing subclones of the primary tumor drive VTT formation.

### Mutational signatures in RCC with VTT

**Figure 5:**
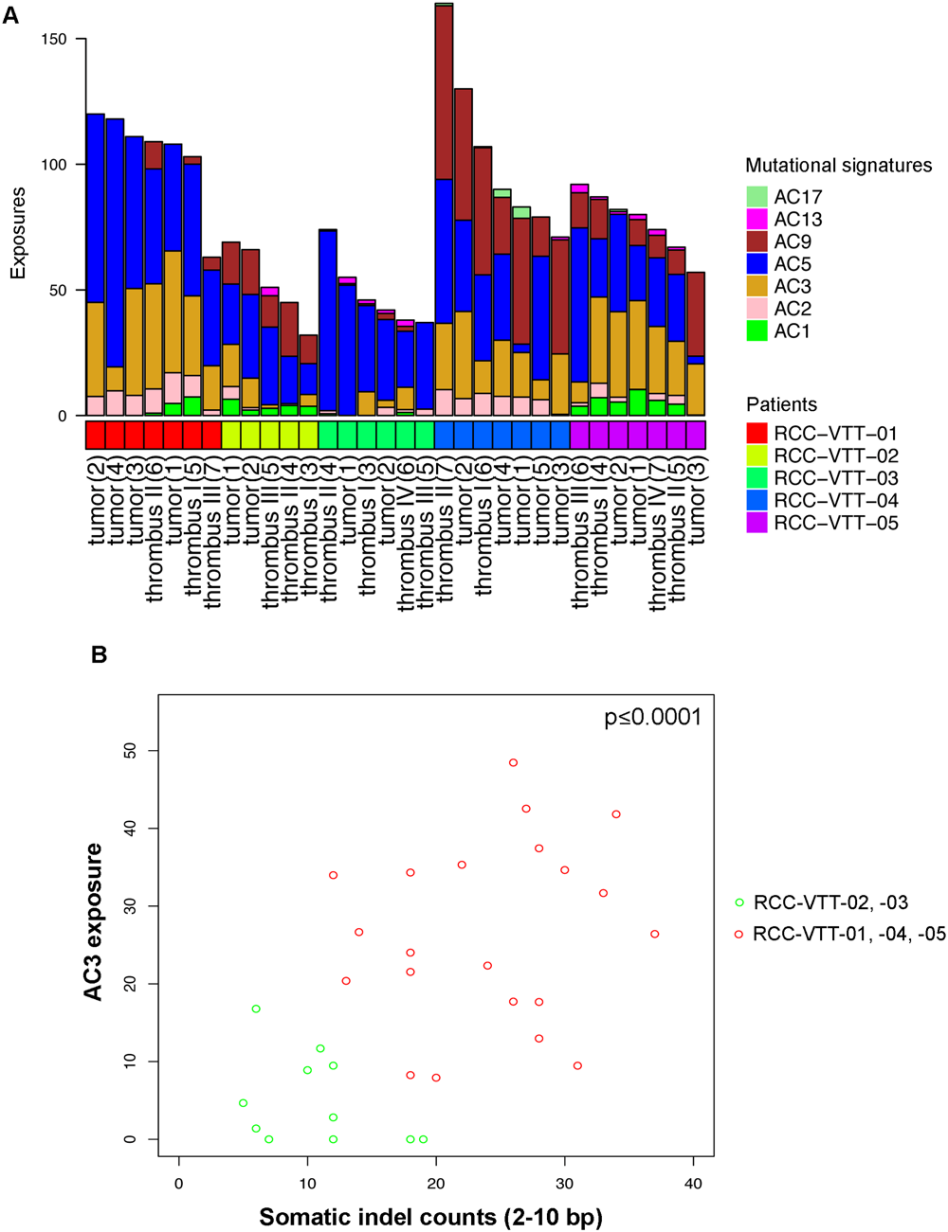
Mutational signatures in RCC with VTT. (**A**) Mutational signature analysis of the five patients. The shown signatures represent the following mutational processes: AC1 = spontaneous deamination; AC2 = APOBEC action; AC3 = homologous recombination repair defect; AC5 = unknown/metabolic stress; AC9 = Pol eta/somatic hypermutation; AC13 = APOBEC; AC17 = unknown. Note the presence of AC3 indicating defective homologous recombination repair in three of the five patients (RCC-VTT-01, -04, -05). (**B**) Scatterplot of exposures to signature AC3 and indel counts (2–10 bp) per sample. Wilcoxon rank-sum test between the two colour-coded groups, p ≤ 0.0001.

Mutational signatures are an emerging concept in understanding cancer genomes and underlying mechanisms of genomic instability^23^. To further elucidate mutational processes in RCC with VTT, we performed a supervised analysis of mutational signatures using the software *YAPSA*^24^, i.e. an analysis with pre-described mutational signatures taken from http://cancer.sanger.ac.uk/cosmic/signatures (Fig. 5A).

Mutational signatures can be grouped into different categories including clock-like signatures^39^, signatures associated with mutagenic enzymatic systems or signatures associated with DNA damage repair defects^23^. In the present cohort and in accordance with previous analyses of mutational signatures in RCC^23,39^, we found a high prevalence of signature AC5 indicating metabolic stress in all samples (Fig. 5A). Moreover, we found exposure to signature AC3 indicating homologous recombination (HR) repair defects in multiple samples of three of the five patients (RCC-VTT-01, -04, -05; Fig. 5A). Signature AC3, which can be caused by mutations in DNA repair genes such as *BRCA1 or BRCA2* but also others, has not been reported in RCC before^23,39^, and may have important translational implications since it is known to confer “BRCAness”^40^. Although no *BRCA1* or *BRCA2* mutations were found in the three RCCs with AC3 exposure, the presence of such mutations is not a prerequisite for “BRCAness”. Instead we detected mutations in *BAP1*, *CDK12*, *ERCC1* or *PTEN*, which have recently been implicated in “BRCAness”^40^. In line with our notion of “BRCAness” in RCC, these genes were found mutated in tumors with mutational signature AC3 (RCC-VTT-01, 04, -05; Fig. 3).

To further corroborate the notion of “BRCAness” in tumors with signature AC3, we analyzed counts of small indels of 2–10 bp length. Small deletions of up to 50 bp have been reported to be characteristic for *BRCA1/2* mutation associated DNA repair defects^41–43^. A significant correlation between exposure to mutational signature AC3 and higher somatic indel counts was detected (Wilcoxon rank-sum test p ≤ 0.0001; Fig. 5B).

In addition, HRD (Homologous Recombination Deficiency) and LST (Large Scale Transition) scores were estimated using smoothed copy number profiles. These two scores, however, did not show a significant correlation with AC3 exposure.

Furthermore, we tested whether indel counts and AC3 exposure were dependent on the localization of the sample taken (i.e., primary tumor or VTT). No such correlation could be corroborated and we conclude that there is no modulating effect of the origin of the specimen in this respect.

**Figure 6:**
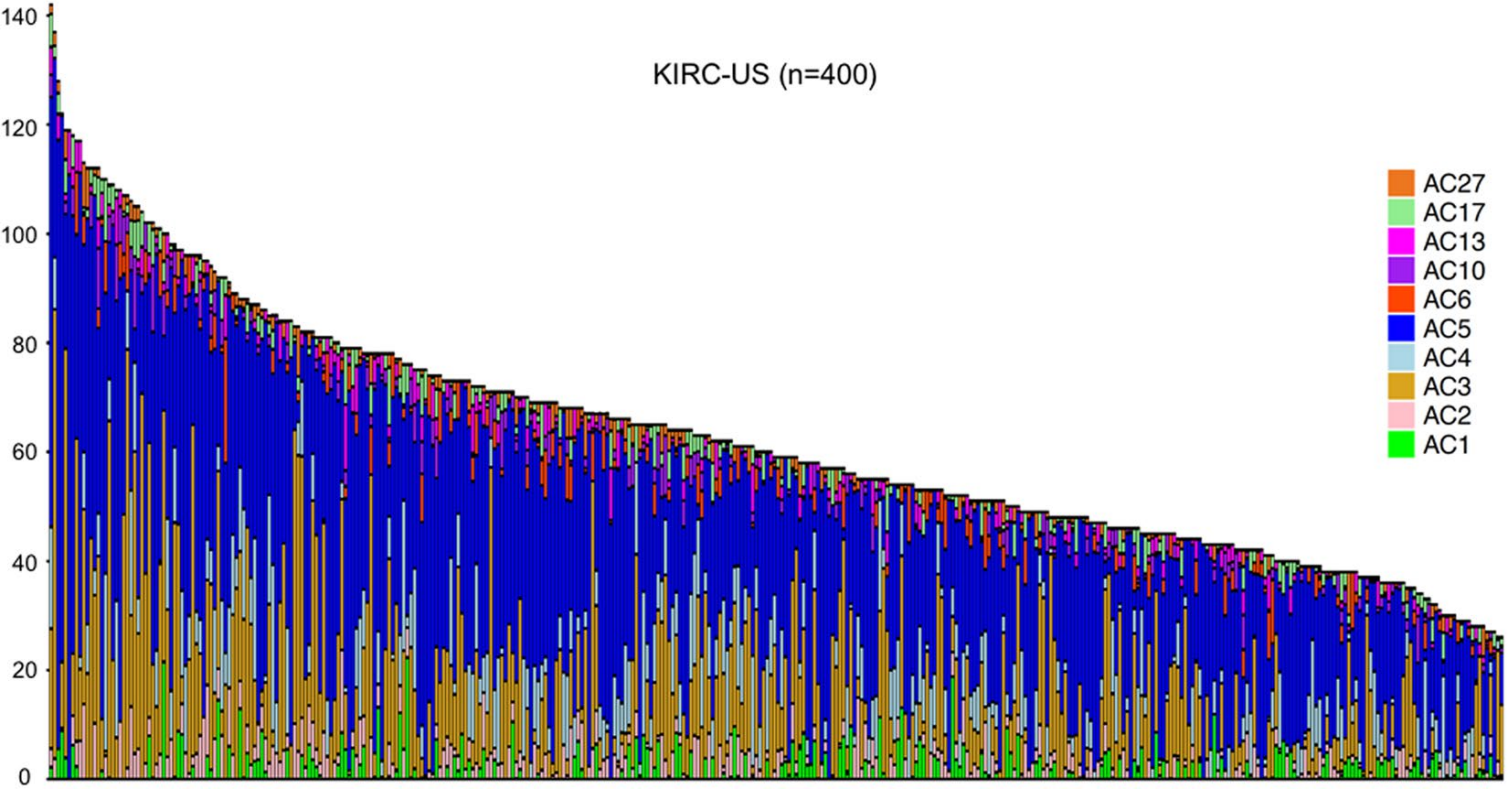
Mutational signatures in the TCGA KIRC-US cohort. Re-analysis of the TCGA KIRC-US cohort using 400 samples of the clear cell RCC TCGA cohort (KIRC-US; patients filtered by a minimum of 25 somatic SNVs per sample; https://portal.gdc.cancer.gov/projects/TCGA-KIRC). Note the high frequency of mutational signature AC3 and AC5. Each bar represents one patient. The y axis depicts the number of mutations. Additional signatures represent the following mutational processes: AC4 = smoking; AC6 = defective DNA mismatch repair; AC10 = altered POLE activity; AC27 = unknown.

Lastly, we sought to determine whether AC3 exposure is a general feature of clear cell RCC. To this end, we re-analyzed data from 400 patients of the clear cell RCC TCGA cohort (KIRC-US, patients filtered by a minimum of 25 somatic SNVs per sample; https://portal.gdc.cancer.gov/projects/TCGA-KIRC) and found AC3 exposure in 297 of 400 samples (74.25%; Fig. 6).

In summary, our results show that a subset of RCCs with VTT show signs of “BRCAness” including exposure to mutational signature AC3 as well as an increase of small indels. A re-analysis of mutational signatures in the TCGA KIRC-US cohort furthermore suggests that AC3 exposure is a frequent finding in clear cell RCC.

## Discussion

Herein, we demonstrate that intravenous tumor thrombi in RCC are not simply an accumulation of tumor cells of uncertain viability and thrombotic material. Instead, our results indicate that they contain viable tumor cells with mutational heterogeneity between primary tumor and VTT. Moreover, we find signs for “BRCAness” in a subset of RCCs with VTT.

The mutational heterogeneity within individual RCCs, among patients and between primary tumors and venous thrombi is in line with previous reports^1,2^. RCCs are also known to harbor more than one driver mutation, a finding that is confirmed in our study^2^. Our results furthermore suggest that, despite mutational heterogeneity, no additional mutations are required for VTT formation and that pre-existing subclones of the primary tumor can drive this process.

“BRCAness” is a relatively recent concept in tumor biology that capitalizes on the fact that defects in error-free DNA double-strand break repair by HR repair represent a common outcome of inactivating mutations in a large number of genes in addition to *BRCA1* or *BRCA2*^15,40^. The mutational signature AC3, which is associated with “BRCAness”, was not found in two large studies on mutational signatures that included RCCs^23,39^. This most likely reflects technical differences since the algorithm to detect mutational signatures used in the present study is supervised and highly sensitive for detecting known mutational signatures. A re-analysis of the TCGA KIRC-US data confirmed a relatively high frequency of signature AC3 in clear cell RCC. Although we were not able to retrieve information on the VTT status in these tumors, the fact that over 70% of tumors showed AC3 exposure in comparison to the reported VTT frequency would argue that AC3 exposure is a more general finding in clear cell RCC and not specific for RCCs with VTT.

RCC is not known to frequently harbor mutations in DNA repair genes and is considered largely insensitive to DNA damaging agents. In the present study, we did not find any mutations in the “classical” HR repair genes such as *BRCA1* or *BRCA2* although such mutations do exist in RCC^44^. However, we did find functional SNVs or indels in genes linked to either HR gene expression (*CDK12*) or execution/completion of HR (*PTEN*, *ERCC1*)^40^. We hasten to add that other genes or combinations of genes that harbor functional SNVs and indels identified in our study could also provide a link to the “BRCAness” signature. Hence, a careful interpretation of the causative role of gene mutations is necessary and eventually requires a functional validation.

It is noteworthy that some genomic characteristics that have been found to be associated with “BRCAness”, namely the HRD and LST scores, did not show a correlation with mutational signature AC3 in our study. This finding could be related to the fact that whole exome sequencing was used, which may lead to an underestimation of these LOH-based scores or may reflect a less stringent correlation between signature AC3, “BRCAness” and LST/HRD scores in RCC in comparison to breast or ovarian cancer with germline *BRCA1/2* mutations.

“BRCAness” has been shown in other tumor entities to lead to an enhanced sensitivity to PARP inhibitors and platinum salts^15^. A key translational question that arises from our study is hence whether RCC patients with AC3 exposure and other signs of “BRCAness” will in fact benefit from such therapies.

Another mutational signature that was frequently detected in our samples is AC5. The unique characteristic of ccRCCs to generally downregulate important metabolic pathways and instead rely on a small metabolic network not usually found in other tumors has recently been reported^45^. These metabolic changes were found to be associated with a loss of the *VHL* tumor suppressor on chromosome 3p. In four of five RCCs analyzed in our study, we found histologically a clear cell phenotype and a corresponding loss of chromosome 3p (Suppl. Fig. 2). All of these tumors showed a ubiquitous presence of mutational signature AC5. In only one tumor (RCC-VTT-04), AC5 was undetectable in one of the specimens (tumor (3)) and detected at a low level in another specimen (tumor (1)). This RCC was a papillary RCC type II with the characteristic *SETD2* and *NF2* mutation^33,34^. The fact that AC5 was detectable in this tumor without *VHL* mutation suggests that other mechanisms may contribute to its origin as well. AC5 has been reported to be high in papillary RCC as well as in ccRCC^39^ before and our results confirm this notion. AC5 has also been suggested to represent a clock-like signature^39^. Patient RCC-VTT-04, the patient with papillary RCC type II and heterogeneous AC5 exposure, was the oldest of the five patients analyzed. This suggests that the tissue of origin and the cancer entity have an important modulating effect on and may even outweigh the clock-like nature of AC5. The biological basis of signature AC5, which can be detected in many human cancers, is still poorly understood and future studies are needed to fully understand the role of AC5 as well as other mutational signatures in tumor development and progression^46^. Although it has recently been suggested that mutational signatures can have a certain degree of gender specificity^47^, the study population used here is too small to draw any conclusions in that respect.

Our report on the mutational landscape of RCC with VTT using multiregion whole exome sequencing of the VTT provides important insights into the natural history of this unique growth pattern. Our results are in line with a previous report of heterogeneous mutations in a venous thrombus from a single RCC patient^2^. We provide the evidence for a “BRCAness” signature in RCC, which may have important translational and clinical implication and may lead to novel treatment modalities such as PARP inhibitors, platinum salts and others in a selected group of patients. Pre-clinical studies to test this notion are urgently warranted.

## Electronic supplementary material

Supplementary Information
