# Supplementary material for: Genomic features of renal cell carcinoma with venous tumor thrombus
Source: Sci Rep. 2018 May 10;8:7477. doi: 10.1038/s41598-018-25544-z (PMC5945671; doi:10.1038/s41598-018-25544-z)
Supplement: Supplementary file 1 — Supplementary Information [file 41598_2018_25544_MOESM1_ESM.docx]

**SUPPLEMENTARY INFORMATION**

**Genomic features of renal cell carcinoma with venous tumor thrombus**

Gregor Warsow^1,a^, Daniel Hübschmann^1-3,a^, Kortine Kleinheinz^1,2^, Cathleen Nientiedt^4-6^, Martina Heller^4^, Laura Van Coile^4^, Yanis Tolstov^4^, Lukas Trennheuser^7^, Kathrin Wieczorek^8^, Carine Pecqueux^6,7^, Claudia Gasch^6,7^, Timur Kuru^7*^, Joanne Nyarangi-Dix^6,7^, Gencay Hatiboglu^6,7^, Dogu Teber^6,7^, Sven Perner^9^, Albrecht Stenzinger^8^, Wilfried Roth^8**^, Boris Hadaschik^6,7***^, Sascha Pahernik^6,7****^, Dirk Jäger^5,6^, Carsten Grüllich^5,6^, Anette Duensing^10^, Roland Eils^1,2^, Matthias Schlesner^1*****,b^, Holger Sültmann^11,b^, Markus Hohenfellner^6,7,b^, and Stefan Duensing^4,6,7,12,b^

^a^These authors share first authorship

^b^These authors jointly supervised the study

^1^Division of Theoretical Bioinformatics (B080), German Cancer Research Center (DKFZ), Im Neuenheimer Feld 280, D-69120 Heidelberg, Germany

^2^Department for Bioinformatics and Functional Genomics, Institute for Pharmacy and Molecular Biotechnology (IPMB) and BioQuant, Heidelberg University, Im Neuenheimer Feld 267, D-69120 Heidelberg, Germany

^3^Department of Pediatric Immunology, Hematology and Oncology, University Hospital Heidelberg, Im Neuenheimer Feld 430, D-69120 Heidelberg, Germany

^4^Section of Molecular Urooncology, Department of Urology, University of Heidelberg School of Medicine, Im Neuenheimer Feld 517, D-69120 Heidelberg, Germany

^5^Department of Medical Oncology, University of Heidelberg School of Medicine, National Center for Tumor Diseases (NCT), Im Neuenheimer Feld 460, D-69120 Heidelberg, Germany

^6^Center for Kidney Tumors, National Center for Tumor Diseases and University of Heidelberg School of Medicine, Im Neuenheimer Feld 460, D-69120 Heidelberg, Germany

^7^Department of Urology, University of Heidelberg School of Medicine, Im Neuenheimer Feld 110, D-69120 Heidelberg, Germany

^8^Institute of Pathology, University of Heidelberg School of Medicine, Im Neuenheimer Feld 224, D-69120 Heidelberg, Germany

^9^Pathology of the University Hospital Schleswig-Holstein, Campus Lübeck and the Research Center Borstel, Leibniz Lung Center, Ratzeburger Allee 160, D-23538 Lübeck and Parkallee 1-40, D-23845 Borstel, Germany

^10^University of Pittsburgh Cancer Institute, Cancer Therapeutics Program, 5117 Centre Avenue, Pittsburgh, PA 15213, USA

^11^National Center for Tumor Diseases, German Cancer Research Center, Cancer Genome Research, Im Neuenheimer Feld 460 and German Cancer Consortium (DKTK), D-69120 Heidelberg, Germany

*Present address: Department of Urology, University of Cologne, Kerpener Str. 62, D-50937 Cologne, Germany

**Present address: Institute of Pathology, University of Mainz Medical School, Langenbeckstr. 1, D-55131 Mainz, Germany

***Present address: Department of Urology, University Hospital Essen, University of Duisburg-Essen, Hufelandstr. 55, D-45122 Essen, Germany

****Present address: Department of Urology, Nuremberg Hospital, Paracelsus Medical University, Prof.-Ernst-Nathan-Strasse 1, D-90419 Nuremberg, Germany

*****Present address: Bioinformatics and Omics Data Analytics (B240), German Cancer Research Center (DKFZ), Im Neuenheimer Feld 280, D-69120 Heidelberg, Germany

Running title: BRCAness in renal cancer

^12^Correspondence should be addressed to:

Stefan Duensing, Section of Molecular Urooncology, Department of Urology, University of Heidelberg School of Medicine, Medical Faculty Heidelberg, Im Neuenheimer Feld 517, D-69120 Heidelberg, Germany, Phone: +49-6221-56-6255, Fax: +49-6221-56-7659, E-mail: stefan.duensing@med.uni-heidelberg.de


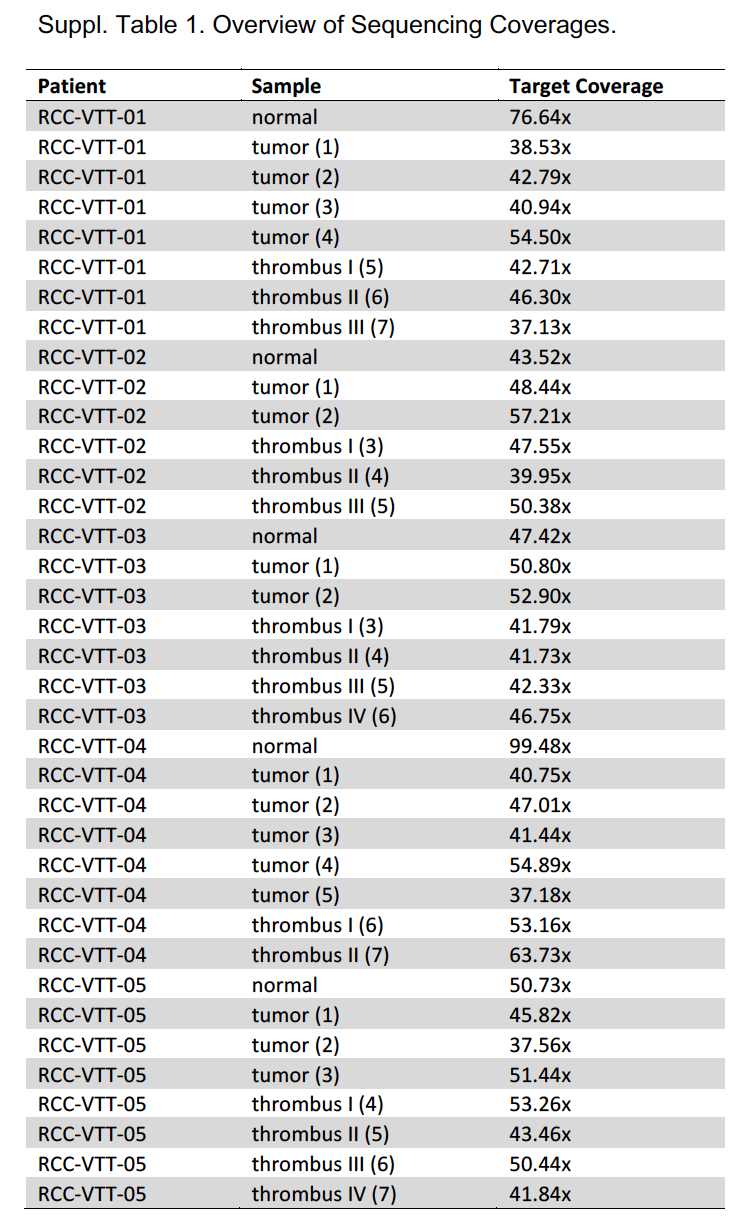


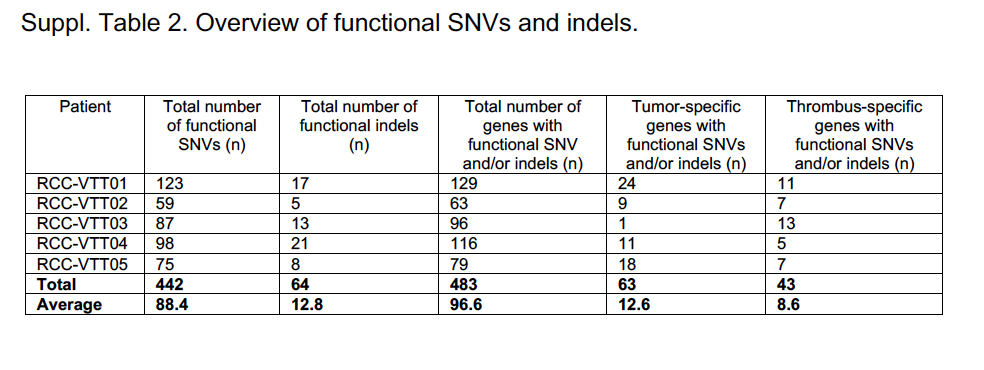


**
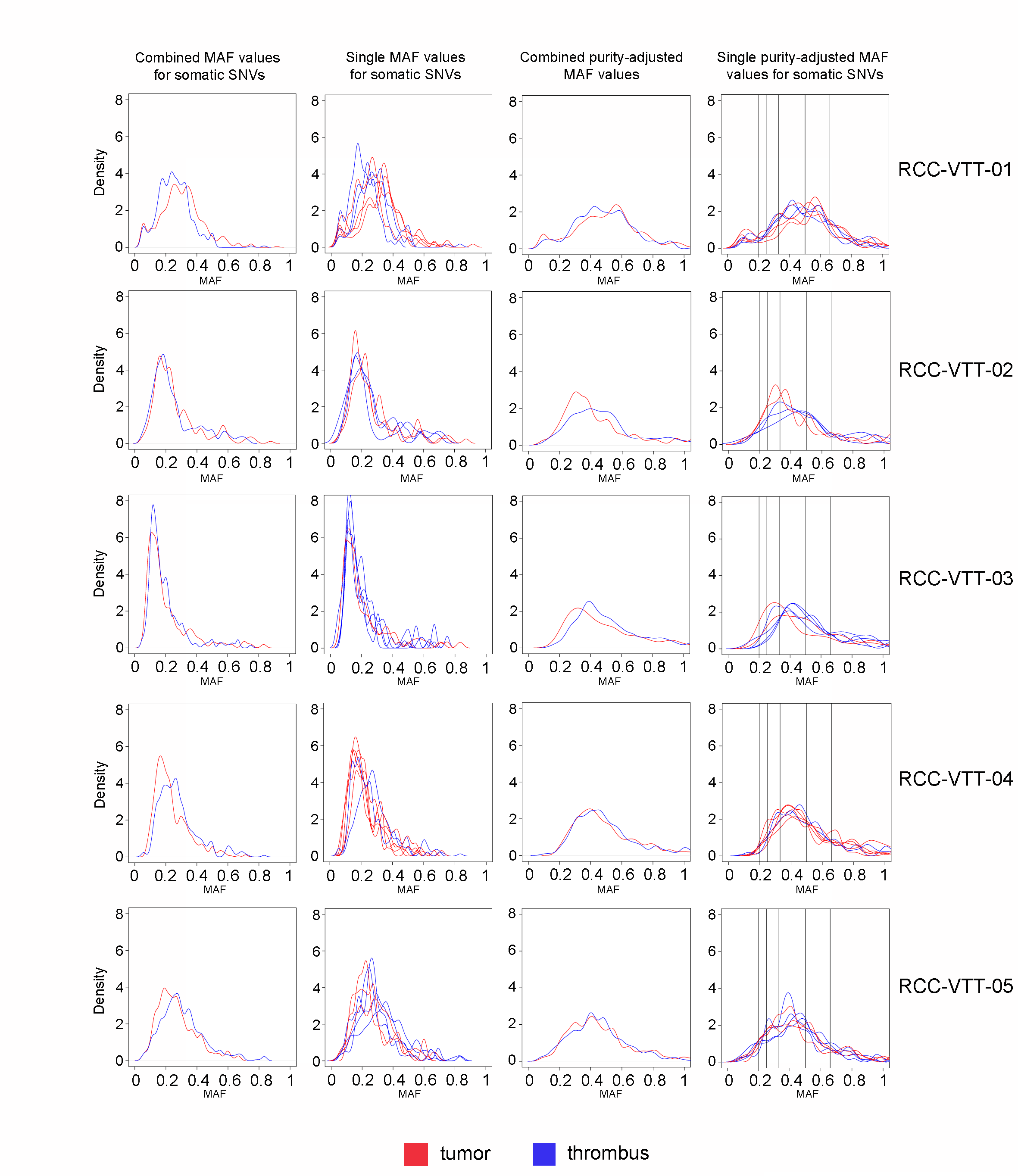
**

**Suppl. Figure 1. MAF distribution in primary tumors and venous tumor thrombi.**

Minor allele frequency (MAF) values for the five patients. Tumor samples are depicted in red and VTT samples in blue. Vertical lines in the single purity-adjusted MAF panels represent the values 0.2, 0.25, 0.33, 0.5 and 0.66.

**
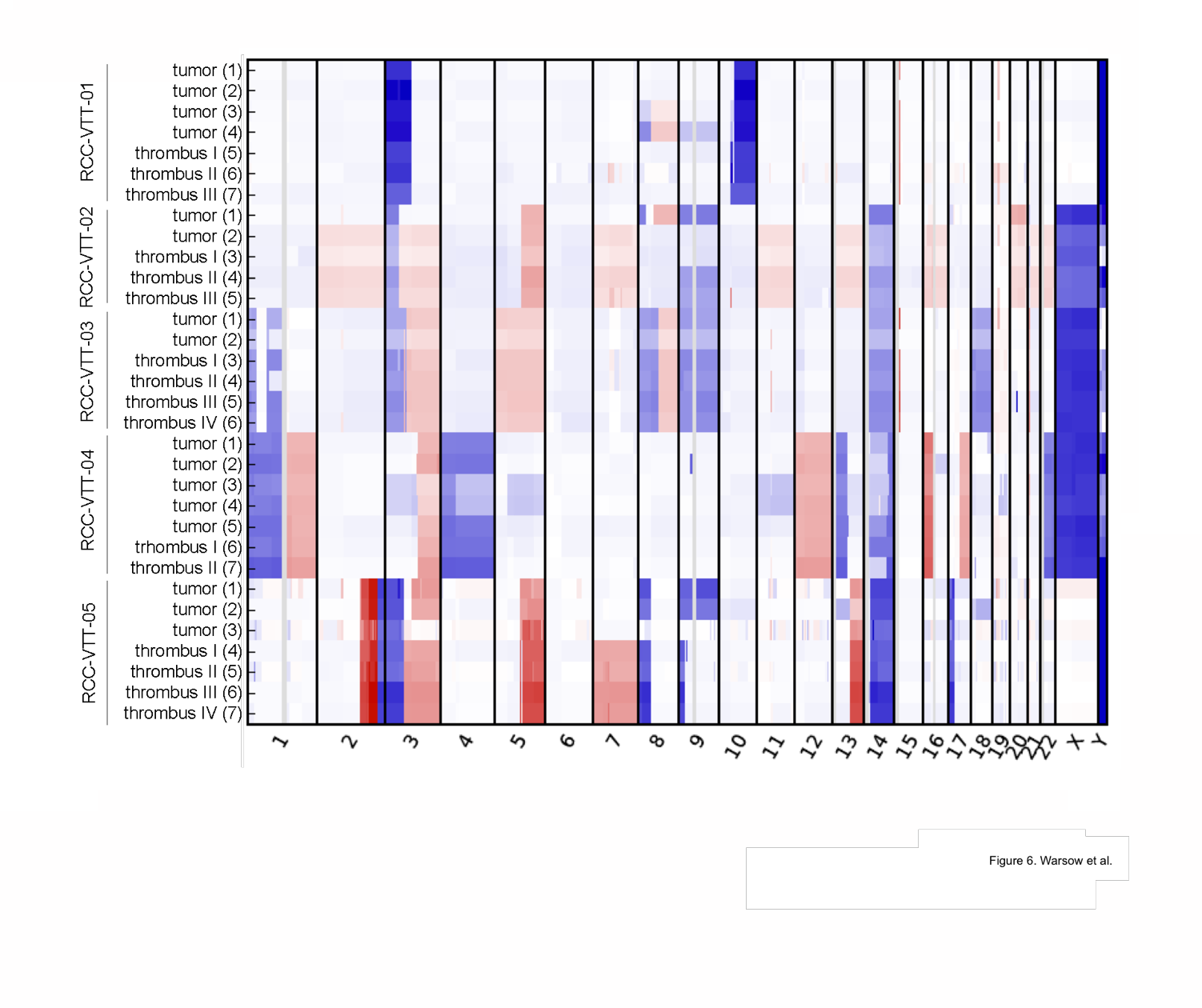
**

**Suppl. Figure 2. Copy number alterations in RCCs.**

Heatmap of chromosome copy number alterations (CNAs) in the 37 samples from five patients with RCC and VTT. Gains are shown in red and losses in blue.

**
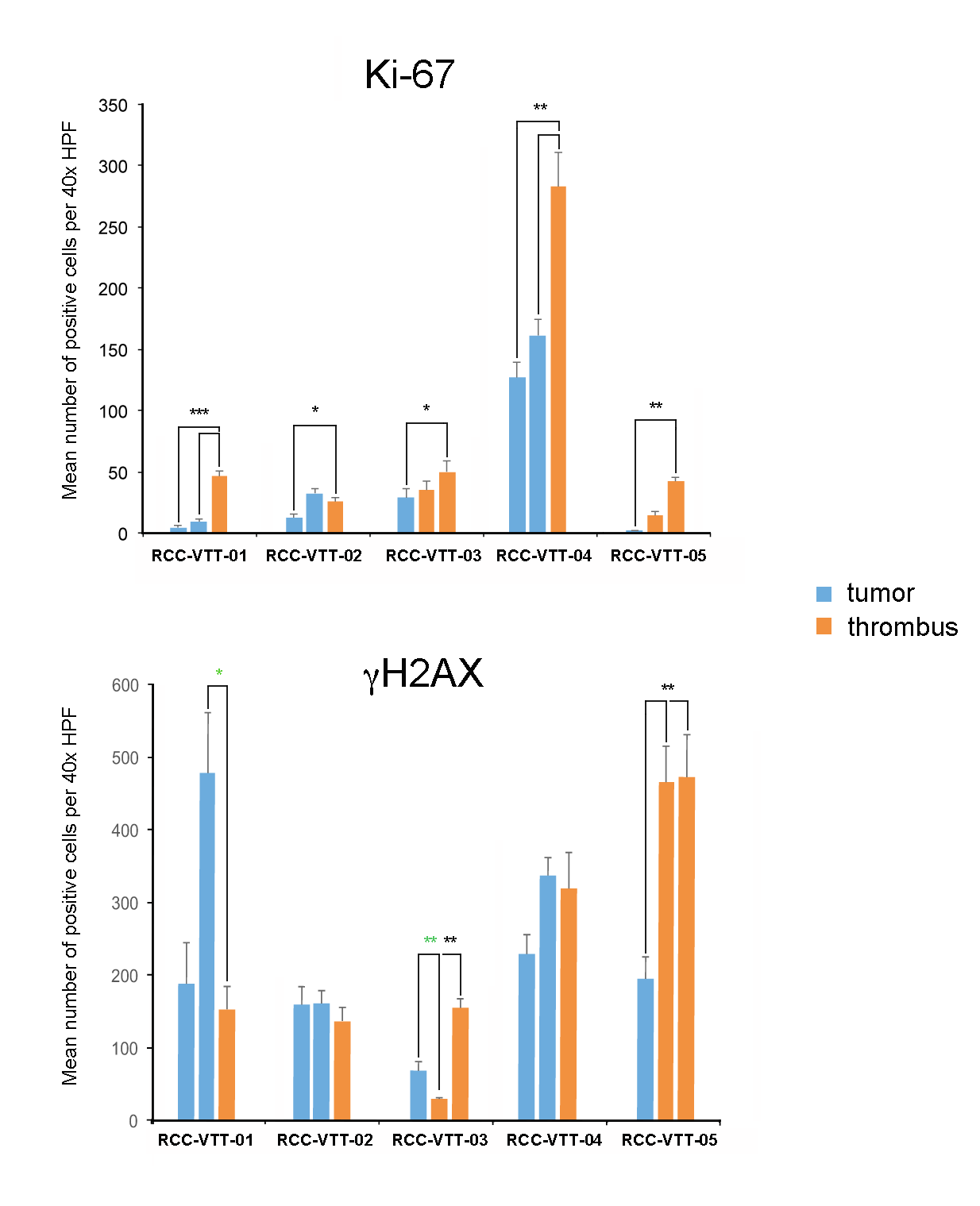
**

**Suppl. Figure 3. Increased tumor cell proliferation in venous tumor thrombi.**

Quantification of Ki-67- or γH2AX-positive tumor cells in representative samples from the primary tumor (blue) or the corresponding thrombus (orange). Mean + S.E. of the number of positive cells per 40x high power field (HPF) are shown. In all patients, at least one thrombus sample showed a significantly higher proliferation rate than one or more primary tumor specimens. Asterisks indicate the level of significance (*p≤0.05; **p≤0.005; ***p≤0.0005). Black asterisks denote a significant increase, green asterisks denote a significant decrease.
